# *In silico* evaluation of DNA Damage Inducible Transcript 4 gene (*DDIT4*) as prognostic biomarker in several malignancies

**DOI:** 10.1038/s41598-017-01207-3

**Published:** 2017-05-08

**Authors:** Joseph A. Pinto, Christian Rolfo, Luis E. Raez, Alexandra Prado, Jhajaira M. Araujo, Leny Bravo, Williams Fajardo, Zaida D. Morante, Alfredo Aguilar, Silvia P. Neciosup, Luis A. Mas, Denisse Bretel, Justin M. Balko, Henry L. Gomez

**Affiliations:** 1 Unidad de Investigación Básica y Traslacional, Oncosalud-AUNA, Av. Guardia Civil 571, San Borja. Lima 41, Peru; 2 Phase I–Early Clinical trials Unit, Antwerp University Hospital & Center for Oncological Research (CORE), Antwerp, Belgium; 3 Thoracic Oncology Program, Memorial Cancer Institute, Memorial Health Care System, Pembroke Pines, FL USA; 4 Escuela de Medicina Humana, Universidad Privada San Juan Bautista, Av. José Antonio Lavalle s/n Hacienda Villa, Chorrillos. Lima 09, Peru; 5 Departamento de Medicina Oncológica, Oncosalud-AUNA, Av. Guardia Civil 571, San Borja. Lima 41, Peru; 6 Grupo de Estudios Clínico Oncológicos Peruano (GECOPERU), Lima, Lima 33 Peru; 7 Departamento de Medicina Oncológica, Instituto Peruano de Enfermedades Neoplásicas, Av. Angamos Este 2520, Surquillo. Lima 34, Peru; 8 Vanderbilt Ingram Cancer Center, Vanderbilt University, Nashville, TN 37232-6307 USA

## Abstract

*DDIT4* gene encodes a protein whose main action is to inhibit mTOR under stress conditions whilst several *in vitro* studies indicate that its expression favors cancer progression. We have previously described that *DDIT4* expression is an independent prognostic factor for tripe negative breast cancer resistant to neoadjuvant chemotherapy. We herein report that high *DDIT4* expression is related to the outcome (recurrence-free survival, time to progression and overall survival) in several cancer types. We performed *in silico* analysis in online platforms, in pooled datasets from KM Plotter and meta-analysis of individual datasets from SurvExpress. High levels of *DDIT4* were significantly associated with a worse prognosis in acute myeloid leukemia, breast cancer, glioblastoma multiforme, colon, skin and lung cancer. Conversely, a high *DDIT4* expression was associated with an improved prognostic in gastric cancer. *DDIT4* was not associated with the outcome of ovarian cancers. Analysis with data from the Cell Miner Tool in 60 cancer cell lines indicated that although rapamycin activity was correlated with levels of *MTOR*, it is not influenced by *DDIT4* expression. In summary, *DDIT4* might serve as a novel prognostic biomarker in several malignancies. *DDIT4* activity could be responsible for resistance to mTOR inhibitors and is a potential candidate for the development of targeted therapy.

## Introduction

*DDIT4* gene (for DNA-damage-inducible transcript 4), also known as *REDD1* or *RTP801*, encodes a protein product that is induced by a variety of stress conditions and whose major function is to inhibit mTORC1 by stabilizing the TSC1-TSC2 inhibitory complex^1–3^.

Despite inhibition of mTOR pathway is a current strategy in the treatment of cancer, paradoxically, several *in vitro* and *in vivo* studies indicate that *DDIT4* have a protective role against apoptosis, where a knockdown of this gene lead to increased levels of dexamethasone-induced cell death in murine lymphocytes without effect in glucocorticoid-induced cell death in primary thymocytes^4, 5^.

A recent study by Celik *et al*., reported that *DDIT4* may be used as a surrogate pharmacodynamic marker of ezrin inhibitors compound activity^6^. Only two previous reports describe the prognostic value of *DDIT4*. Jia *et al*.^7^, evaluated DDIT4 protein expression (assessed by immunohistochemistry) in 100 primary ovarian tumors describing that a high DDIT4 expression is related to a shorter disease-free survival (P = 0.020) and overall survival (P = 0.023)^7^. In the other hand, our group screened 449 genes related with triple negative breast cancer aggressiveness and found that a high *DDIT4* expression was an independent factor associated with a shorter disease-free survival in chemotherapy-resistant triple negative breast tumors (HR = 1.56 by each unit of change; P = 0.005)^8^.

Although some mTOR inhibitors have approval for several malignancies, none study have shown that mTOR expression itself is a predictive or prognostic factor; conversely, several resistance mechanisms develops in cancer cells limiting the use of mTOR inhibitors^9, 10^.

Due to the need of exploring new targets to overcome resistance to mTOR inhibitors new related targets should be evaluated where modulation of DDIT4 activity could be a promising therapeutic strategy. Yang *et al*.^11^, described that in β cells, inhibition of DDIT4 by high glucose media increases expression of apoptosis regulating proteins, such as phospho-Bcl-2, cytochrome C and cleaved caspase^11^. In addition, ectopic *DDIT4* expression in Müller cells was sufficient to VEGF expression in the murine model suggesting a potential role in tumor angiogenesis^12^. All this data suggest a driver role for *DDIT4* in the aggressiveness of cancer cells.

In this work we analyzed publicly available online datasets with the purpose of evaluate *DDIT4* expression as possible biomarker in the outcome of several tumor types.

## Results

### Study characteristics

The prognostic value of *DDIT4* was evaluated in online platforms (KM-Plotter and SurvExpress) in several cancer types. The list of cancers types and datasets evaluated are listed in Table S1.

### Structural alterations of *DDIT4* in various cancers

Overall, data from distinct available genomic projects in cBioPortal showed a low prevalence of structural alterations in *DDIT4*. In malignant breast tumors DDIT4 mutations had frequencies ranging: 0.4–1.5% in primary breast tumors (mainly amplifications). In contrast, 17% of breast cancer xenografts present amplifications. The higher prevalence was observed in pancreatic neuroendocrine tumors, were 10% of mutations were found (1 out 10 cases). In prostate cancer, frequency of amplification occurs between 0.3–8.7% (Figure S1).

### Acute Myeloid Leukemia (AML)

**Figure 1:**
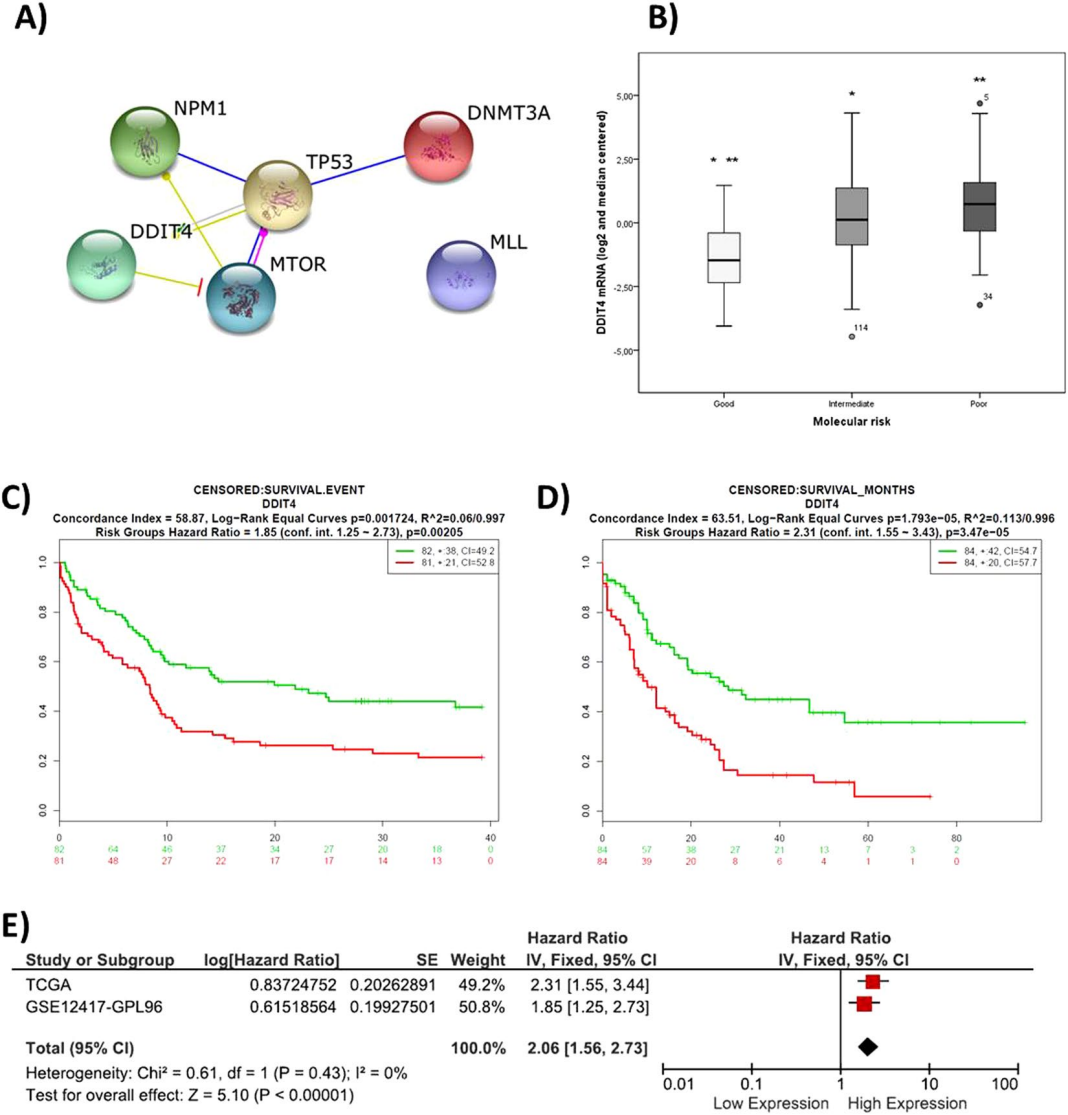
Evaluation of DDIT4 in the survival of AML patients. (**A**) Protein interaction of DDIT4 and genes related with the outcome in AML. (**B**) DDIT4 is associated with the molecular risk in AML patients (P < 0.001). Survival analysis of AML patients stratified by DDIT4 expression in datasets contained in SurvExpress show that high DDIT4 expression (over the median) is associated to a poor prognosis in the (**C**) TCGA dataset [N = 168] and in (**D**) GSE12417−GPL96 dataset [N = 168]. (**E**) A meta-analysis in these two datasets shows 2.6 times increasing in death risk in AML patients with high DDIT4 expression.

Protein-protein interaction of DDIT4 with proteins encoded by genes related with good prognosis in AML, as predicted in The STRING database v. 10 (http://string-db.org/)^13^ indicates that DDIT4 and NPM1 have interaction with mTOR and p53; in the other hand, DDIT4 and DNMT3A interact with p53 (Fig. 1A). Analysis of the TCGA data for AML shows that *DDIT4* expression is directly correlated with the molecular risk (P < 0.001) (Fig. 1B). SurvExpress contained only two datasets with overall survival (OS) data (TCGA, N = 168 and GSE12417-GPL96, N = 168). A high *DDIT4* expression (above the mean) was associated with a poor prognosis in both datasets with a HR = 1.85 (P = 0.00205, 95% CI: 1.25–2.73) for the TCGA dataset (Fig. 1C), and an HR = 1.55 (P = 3.47e-05, 95%CI: 1.55–3.43) for GSE12417-GPL96 (Fig. 1D). A meta-analysis of these datasets was done, obtaining a total HR = 2.06 (P < 0.00001, 95% CI: 1.56–2.73). There was no evidence of statistical heterogeneity (P = 0.43) between datasets (Fig. 1E).

### Breast Cancer

**Figure 2:**
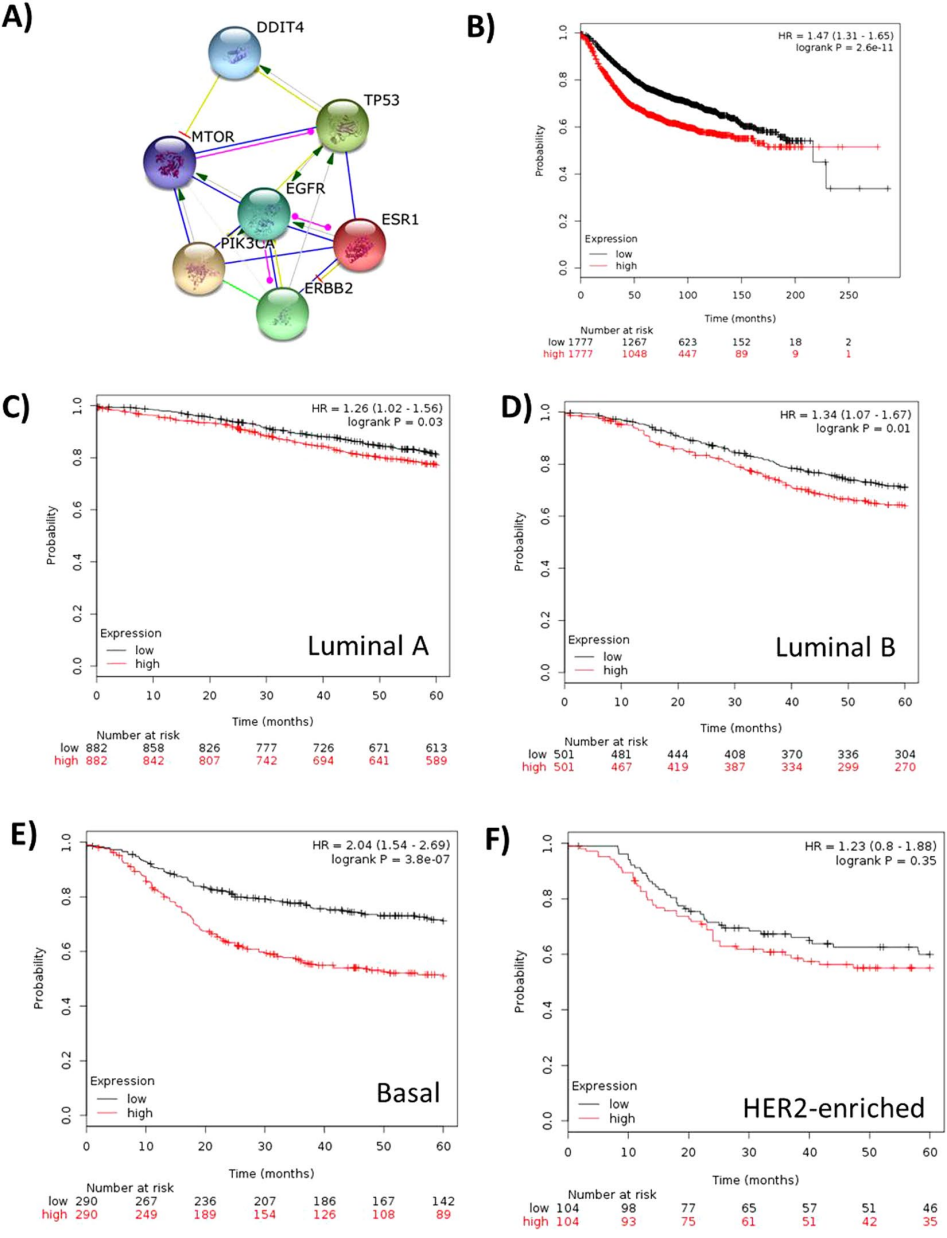
Evaluation of DDIT4 expression in the survival of breast cancer patients. (**A**) Protein interaction of DDIT4 with genes relevant in breast cancer. (**B**) Analysis in KM-Plotter shows that high DDIT4 expression is related with a poor prognosis [P = 2.6 × 10 -11]. In 5-years censored data, (**C**) DDIT4 was a prognostic factor in Luminal A [P = 0.003], (**D**) Luminal B [P = 0.001] and (**E**) Basal subtype [P = 3.8 × 10 -7]. (**F**) DDIT4 was not related with the outcome in patients with HER2-enriched tumors.

Prediction of interaction of DDIT4 protein with relevant gene products in breast cancer indicated convergence in mTOR and p53 (Fig. 2A). Evaluation of *DDIT4* value in recurrence-free survival (RFS) in 3554 patients from KM Plotter (Affymetrix probe ID: 202887_s_at), showed that high *DDIT4* expression is related with a poor prognosis (HR = 1.47; P = 2.6e-11, 95%CI: 1.31–1.65) (Fig. 2B). When the pooled dataset was stratified according to the molecular subtype of breast cancer, the logrank test indicated that *DDIT4* expression over the median was significantly related with a poor prognosis in Luminal A (P = 0.03) (Fig. 2C); Luminal B (P = 0.01) (Fig. 2D) and in the Basal subtype (P = 3.8 × 10^−7^) (Fig. 2E). However, *DDIT4* was not related with the RFS in HER2-enriched tumors (P = 0.35) (Fig. 2F). On the other hand, *DDIT4* evaluation in the SurvExpress platform showed that *DDIT4* expression was related to a poor prognosis (in terms of RFS) in 3 out of 15 breast cancer datasets (Vant Veer Nature, GSE4922 and GSE19615). A meta-analysis in 15 datasets indicated relationship with the outcome, where a *DDIT4* expression over the median increases the recurrence risk in 24% (P = 0.0006, 95%CI: 1.24–1.40). There was no statistical heterogeneity between datasets (P = 0.20) (Figure S2). *DDIT4* expression was associated with the OS in datasets contained in KM-Poltter (Figure S3A) and SurvExpress platforms (Figure S3B).

### Glioblastoma

**Figure 3:**
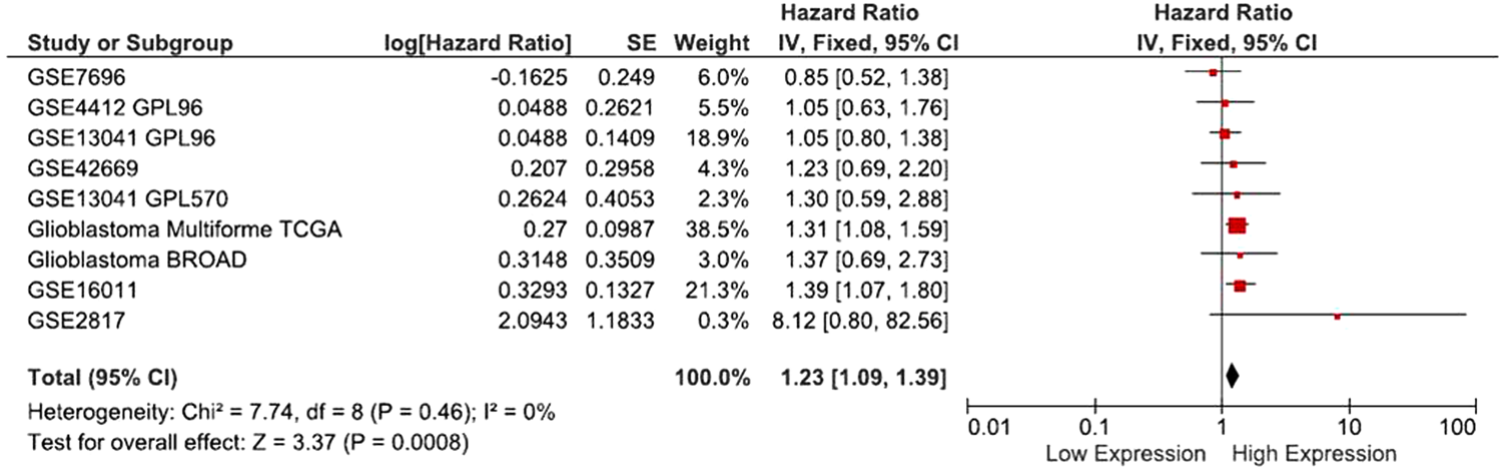
Meta-analysis of 9 glioblastoma SurvExpress datasets, showing that a DDIT4 expression over the median increases the risk of death by 23%.

SurvExpress platform had 9 glioblastoma datasets where *DDIT4* overexpression was related with an increased risk of death in 2 out of 9 datasets (TCGA dataset for glioblastoma multiforme and GSE16011). The meta-analysis in all datasets showed that *DDIT4* overexpression a 23% increased risk of death (P = 0.0008; CI95%: 1.09–1.39). There was no clear evidence of statistical heterogeneity (p = 0.46) between datasets (Fig. 3).

### Ovarian cancer

Analysis of RFS in KM-Plotter was done in a pool of 13 datasets. *DDIT4* overexpression confers an 18% increase of risk of recurrence (HR = 1.18; CI95%: 1.03-1-34) with a P-value = 0.015 in the logrank test (Figure S4A). The RFS analysis with 5-years censored data indicates a 20% increase risk of recurrence (HR = 1.2; CI95%: 1.04–1.37; P = 0.0096) (Figure S4B). A meta-analysis in 6 datasets from SurvExpress show not significant association between DDIT4 and the overall survival (HR = 1.14; CI95%: 1.00–1.31; P = 0.05) (Figure S4C).

### Gastric Cancer

**Figure 4:**
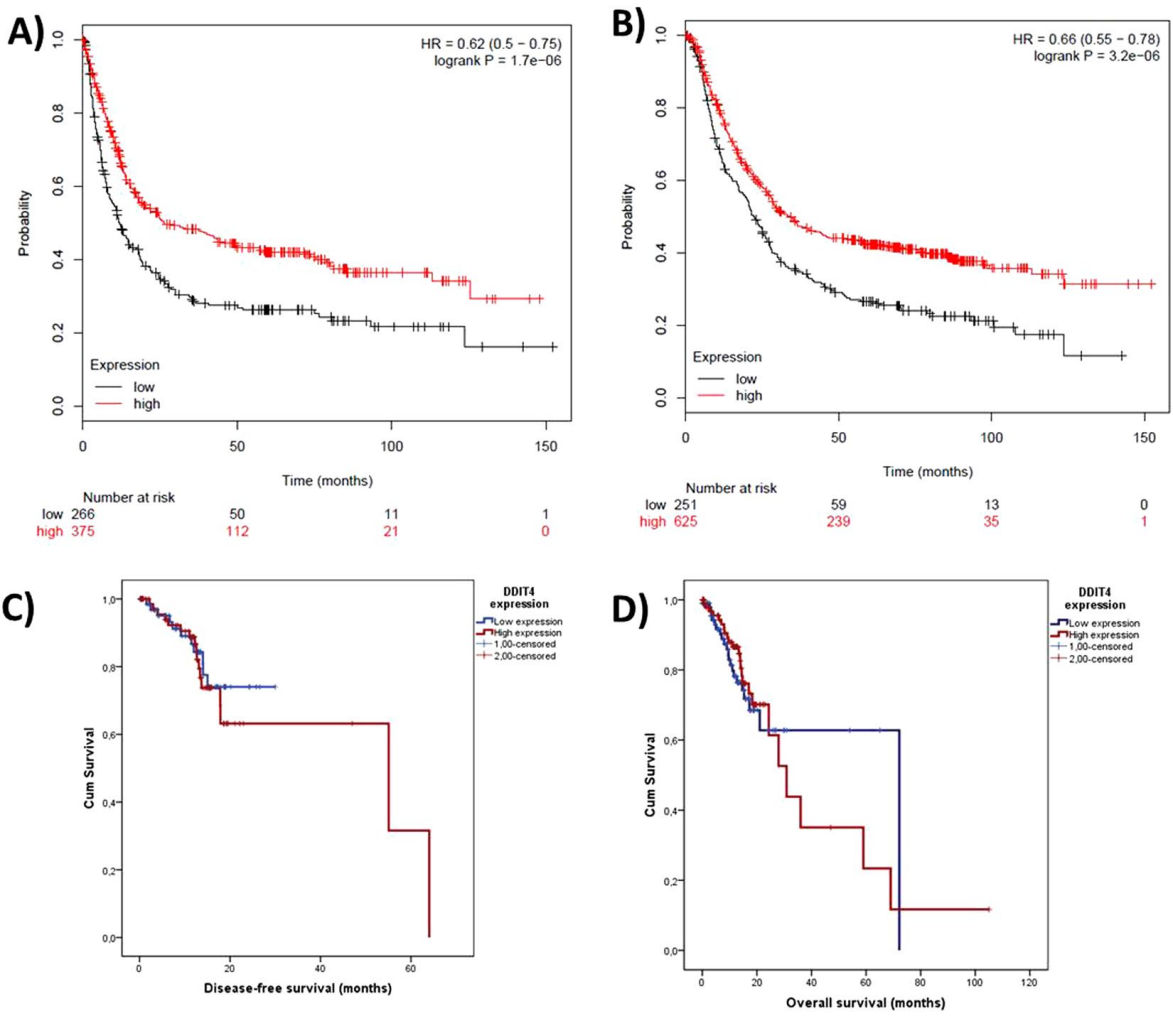
Analysis of pooled gastric cancer datasets contained in KM-Plotter. It showed that a high DDIT4 expression is related with a good prognosis in (**A**) time to first progression [N = 646; P = 1.7 × 10−6] and in (**B**) Overall survival [N = 876; P = 3.2 × 10−6]. However; data of gastric adenocarcinoma downloaded from the TCGA project show not association between DDIT4 expression and the (**C**) disease-free survival [N = 148; P = 0.870] or (**D**) overall survival [N = 208; P = 0.850].

Analysis in KM-Plotter in a pool of 7 datasets shows that a *DDIT4* expression over the median is a protective factor for time to first progression (HR = 0.62; CI95%: 0.5–0.75, with a P-value in the logrank test of 1.7 × 10^−6^) (Fig. 4A) and for OS (HR = 0.66; CI95%: 0.55–0.78, with a P-value in the logrank test of 3.2 × 10^−6^) (Fig. 4B). The TCGA dataset of gastric adenocarcinoma in SurvExpress was not evaluated due to it have only 5 deaths events registered. However, the analysis of the data downloaded from the TCGA for gastric adenocarcinoma evidenced not differences in survival when group of patients was split into two groups according to *DDIT4* expression (P-value in the logrank test of 0.999) (Fig. 4C).

### Lung cancer

**Figure 5:**
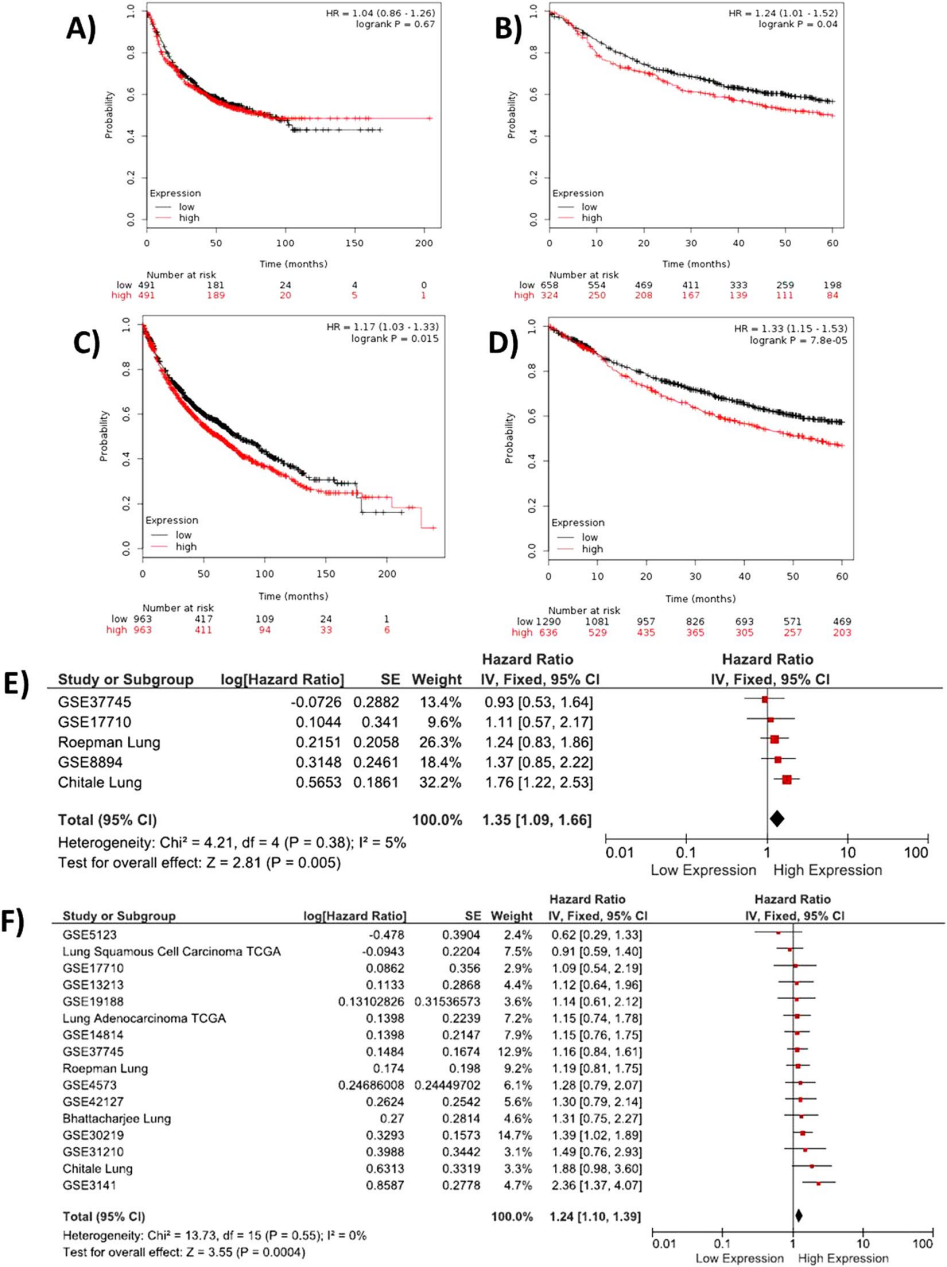
Analysis of pooled datasets of lung cancer. (**A**) Evaluation in KM-plotter shows that DDIT4 expression (cutoff over the median) is not related with the free progression time; however (**B**) in 5-years censored data stratified in the upper tertile, high DDIT4 expression is related with PF [N = 982; P = 0.04] (**C**) Overall survival analysis show that DDIt4 expression over the median is related with a poor outcome (P = 0.015), (**D**) increasing the significance when data is 5-years censored and stratified by the upper tertile. Meta-analysis in SurvExpress show DDIT4 expression over the median increases the (**E**) risk of recurrence by 35% and (**F**) the risk of death by 24%.

Analysis of 14 datasets pooled in Km-Plotter shown no correlation between progression-free survival with *DDIT4* expression (higher vs lower the median) (P = 0.67) (Fig. 5A); however, when data was 5-years censored and the dataset was split at the upper tertile of *DDIT4* expression, a significant association was observed (P = 0.04) (Fig. 5B). In the other and *DDIT4* expression over the median was associated with a shorter OS (P = 0.015) (Fig. 5C) and when the entire cohort is 5-years censored and divided in the upper tertile, the significance increases (P = 7.8 × 10^−5^) (Fig. 5D). The meta-analysis of datasets contained at SurvExpress indicates that *DDIT4* overexpression is associated with a risk of recurrence increase of 35% (P = 0.005) (Fig. 5E) and a risk of death increase in 24% (P = 0.0004) (Fig. 5F).

### Melanoma

The meta-analysis of 3 datasets from SurvExpress showed an 94% increased risk for death (P = 0.006; CI95%: 1.21–3.10) for patients with a *DDIT4* overexpression. Only one dataset (GSE22153) showed association between *DDIT4* and OS. There was no clear evidence of statistical heterogeneity between datasets (P = 0.43) (Figure S5).

### Colon cancer

**Figure 6:**
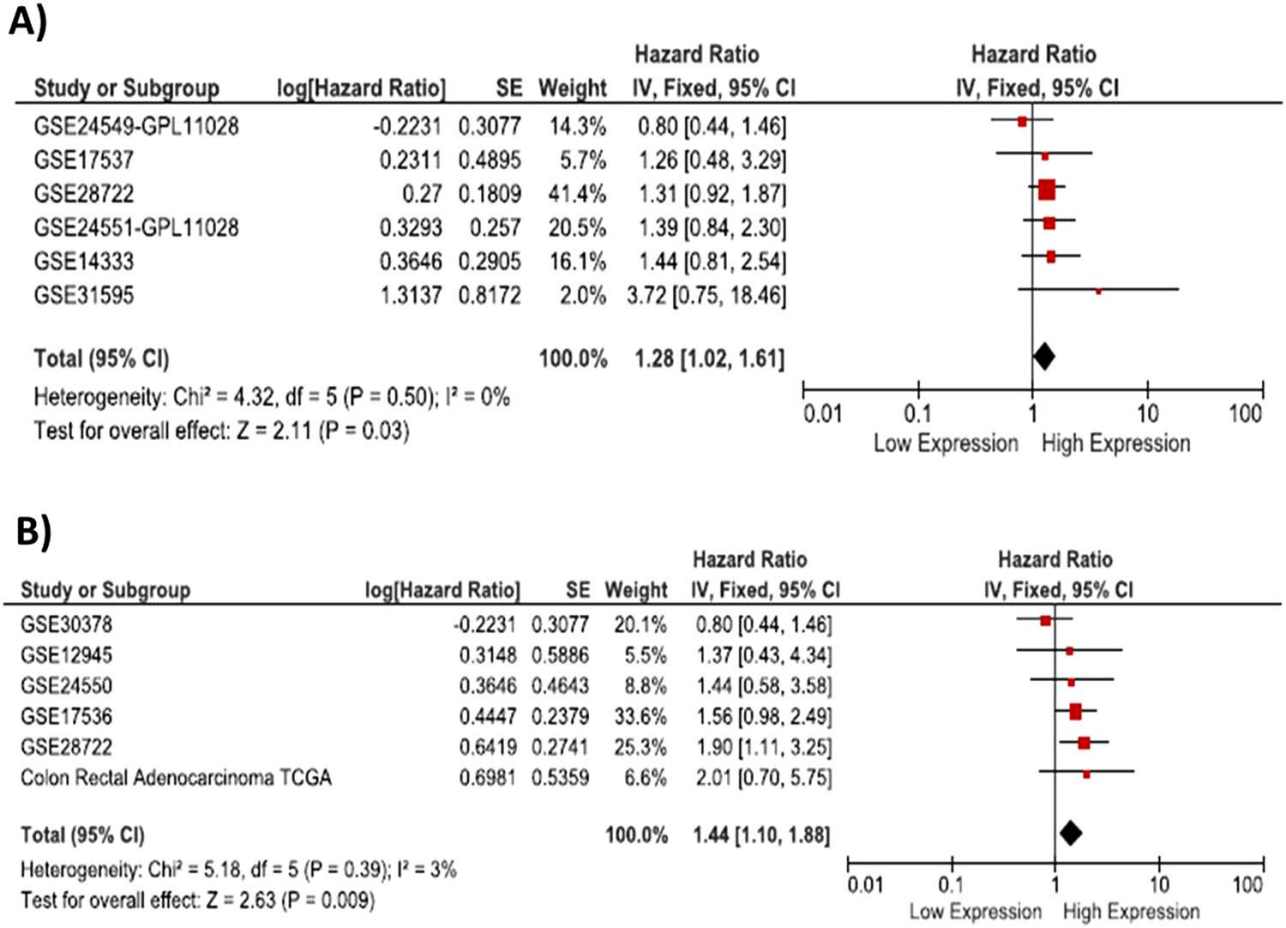
Meta-analysis of colon cancer datasets contained in SurvExpress. (**A**) A DDIt4 overexpression increases the risk of recurrence by 28% (P = 0.03) (**B**) and the risk of death by 44% (P = 0.009).

The meta-analysis of 6 datasets contained in SurvExpress show a HR = 1.28 for recurrence (CI95%: 1.02–1.61; P = 0.03) for patients with tumors expressing *DDIT4* over the median although no dataset has significant association (Fig. 6A). The meta-analysis for OS indicates a HR = 1.44 for patients with a *DDIT4* expression over the median (CI95%: 1.10–1.88; P = 0.009). Only one dataset (GSE28722) had a significant association (Fig. 6B).

### Liver, kidney, bladder, head and neck and prostate cancers

Meta-analysis in SurvExpress shows not significant association between DDIT4 expression with the outcome when patients were grouped using the median of DDIT4 expression as a cutoff. In liver cancer the meta-analysis results in a HR = 1.10 (CI95%: 0.90–1.51; P = 0.55) for RFS and in a HR = 1.12 (CI95%: 0.87–1.44; P = 0.38) for OS (Figure S6). In Kidney cancer, the resulting HR for OS was 1.19 (CI95%: 0.94–1.51; P = 0.14) (Figure S7). For bladder cancer is observed a HR = 1.35 for OS (CI95%: 0.98–1.87; P = 0.07) (Figure S8). In head and neck cancer, the meta-analysis for OS resulted in a HR = 1.32 (CI95%: 0.87–2.00) (Figure S9). For prostate cancer, the meta-analysis for OS resulted in a HR = 1.30 (CI95%: 0.81–2.11) (Figure S10).

### Drug activity of rapamycin is not influenced by DDIT4 expression

**Figure 7:**
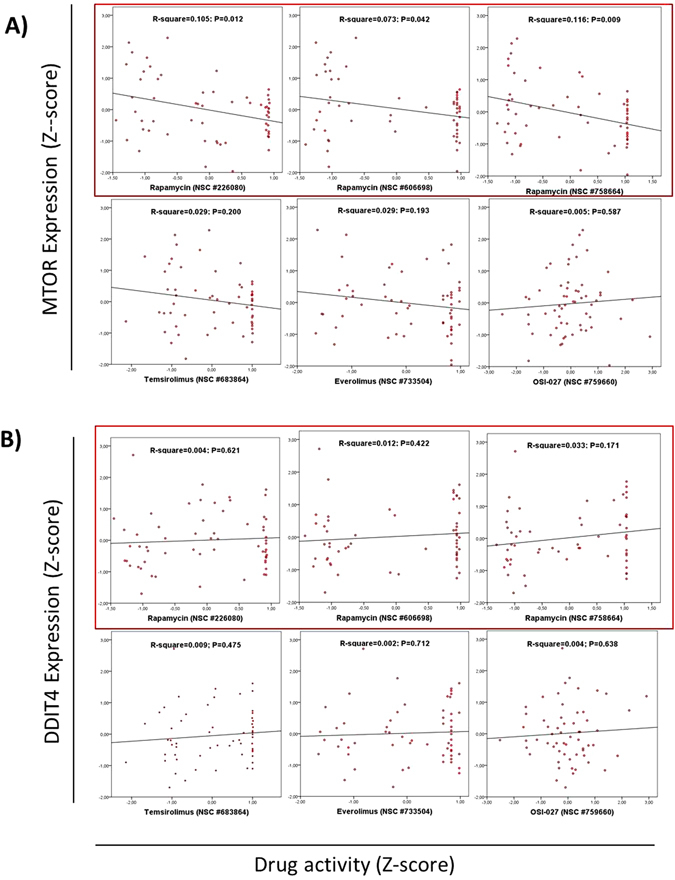
Evaluation of the influence of DDIT4 expression in drug activity of rapamycin. Drug activity of mTOR inhibitors is not influenced by DDIT4 expression in an analysis of 60 cancer cell lines evaluated in Cell Miner Tool. (**A**) mTOR expression is only related to rapamycin response (in a red box). (**B**) DDIT4 is not related with the response to rapamycin (red box) or to the mTOR inhibitors.

We downloaded data from the Cell Miner Analysis Tool project (http://discover.nci.nih.gov/cellminer/) in order to know if drug activity in mTOR inhibitors including rapamycin, everolimus, temsorolimus and OSI-127 is influenced by DDIT4 expression in 60 cancer cell lines. mTOR expression was correlated only with drug activity of rapamycin (Fig. 7A). *DDIT4* expression was not correlated with drug activity of rapamycin (Fig. 7B).

### DDIT4 expression but not PI3K/mTOR pathway alterations is related to a poor outcome in TNBC

**Figure 8:**
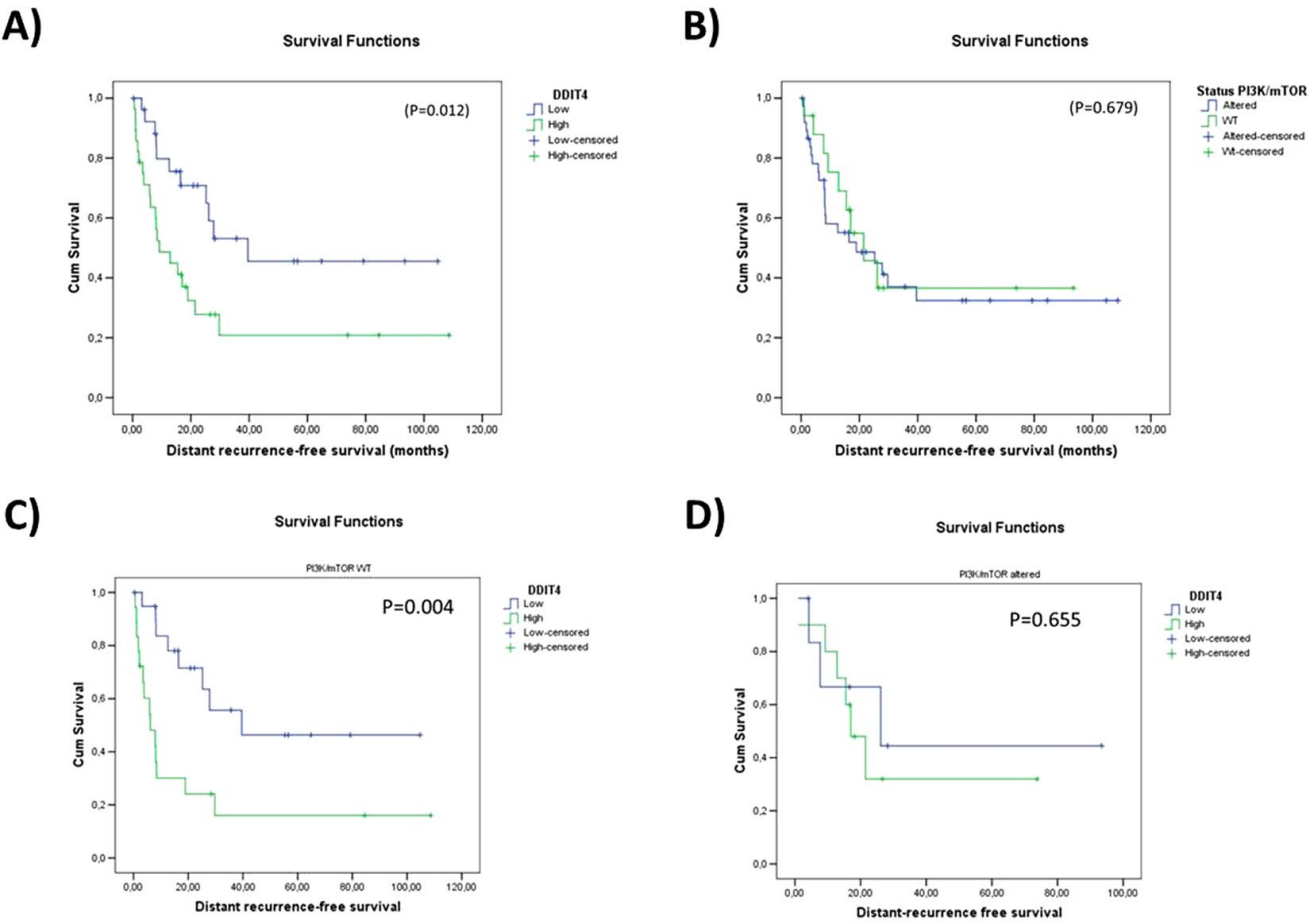
Evaluation of DDIT4 expression and PI3K/mTOR pathway alterations in TNBC. Kaplan-Meier plots for DRFS in a cohort of 58 TNBC patients. (**A**) Patients with a high expression of DDIT4 had a worse outcome (P = 0.012). (**B**) PI3K/mTOR pathway alterations had no influence in the outcome (P = 0.679).  When the cohort was split according to the PI3K/mTOR pathway status, differences in survival was observed in patients with unaltered PI3K/mTOR pathway (**C**), in patients with altered PI3K/mTOR pathway was not observed differences (**D**).

We evaluated the influence of DDIT4 and genomic alterations of PI3K/mTOR pathway in the outcome of 58 TNBC samples with matched DDIT4 expression data (assessed with Nanostrings) and sequencing data for AKT1, AKT2, AKT3, PIK3CA, RAPTOR, RICTOR, PTEN, TSC1, PIK3CA and PIK3R1 genes. Patients with a DDIT4 expression over the median had a worse outcome in terms of distant-recurrence free survival (P = 0.012) (Fig. 8A). In the other hand, there were not differences when patients were stratified according to alterations in the PI3K/mTOR pathway (P = 0.679) (Fig. 8B). When the cohort was divided according the PI3K/mTOR pathway status, a high DDIT4 expression was associated with a shorter distant-metastases free survival only in patients without PI3K/mTOR pathway alterations (Fig. 8C and D).

## Discusion

Metabolism of malignant tumors has been widely studied because it’s an attractive therapeutic target to disrupt cancer cell proliferation^14^. Although mTOR pathway inhibition is a current targeted therapy strategy, several *in vitro* and *in vivo* studies have shown that *DDIT4* expression could lead to cancer progression, resistance to treatment and angiogenesis, raising an important question about the mTOR biology.

**Figure 9:**
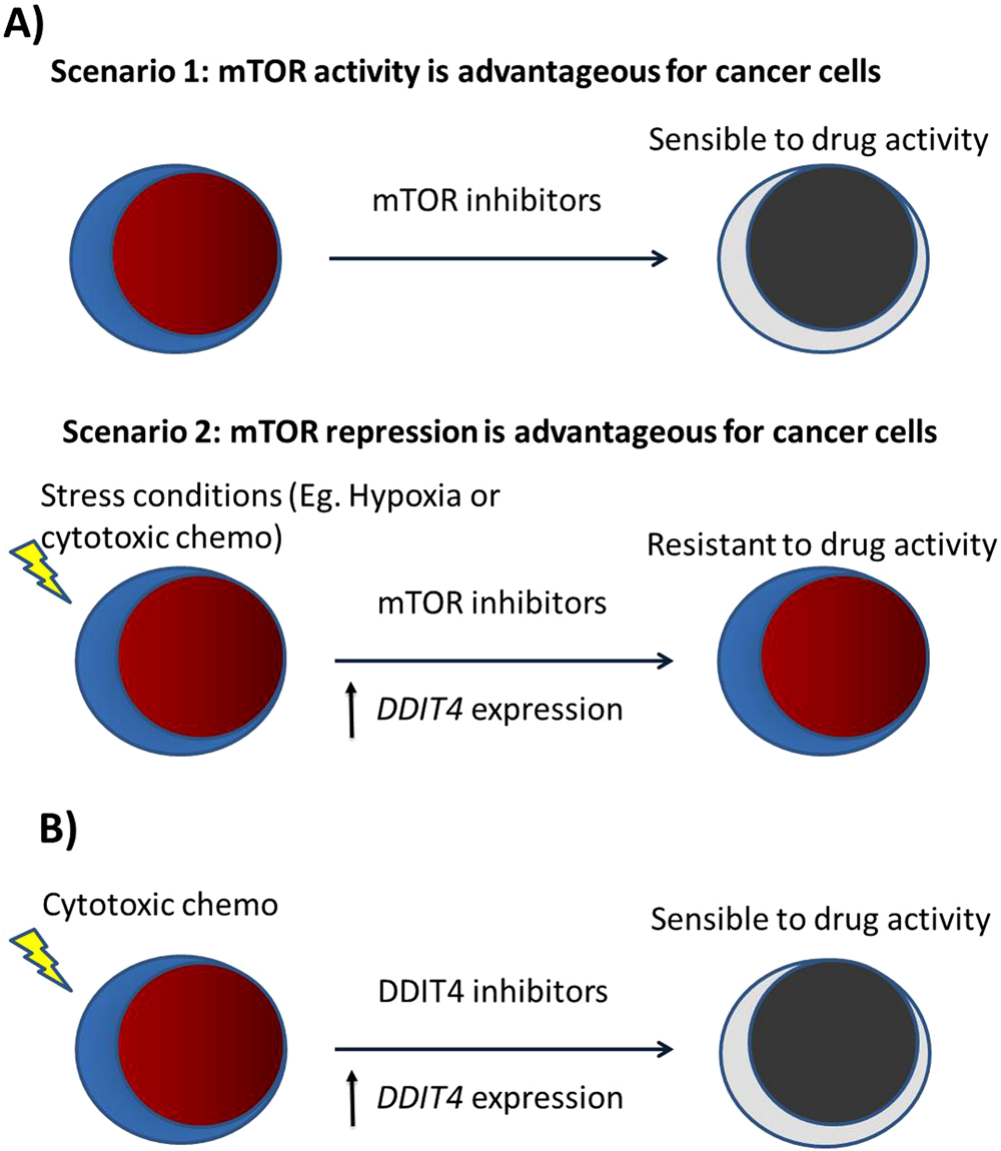
Proposed scenarios of DDIT4 activity. (**A**) Efficacy of mTOR inhibitors depends of the context or “scenario” according to status of activation/repression of mTOR. Resitance to mTOR inhibitors could be explained by the advantageous situation when DDIT4 repress tumor cells under stress conditions. (**B**) Overcome of resistance could be achieved with combination of cytotoxic chemotherapy and DDIT4 inhibitors.

We think that under normal physiological condition mTOR is an important player for tumor aggressiveness and inhibition of mTOR pathway results in an effective therapeutic strategy. However, under cellular stress conditions (such as hypoxia or cytotoxic chemotherapy), mTOR activity is disadvantageous for cancer cells and the suppression of mTOR activity by *DDIT4* is important for tumor survival. This fact could suggest that non cytotoxic drugs (as letrozole in breast cancer, for example) are better combinations of mTOR inhibitors than cytotoxic chemotherapy (Fig. 9). In our TNBC model, high *DDIT4* expression but not PI3K/mTOR pathway alterations was predictor of shorter survival (Fig. 8).

In this work we show that *DDIT4* expression is related with the outcome in multiple cancer types where patients whose tumors expressed *DDIT4* over the median had a >20% increment in the risk of relapse or death. Although it could be a modest increase in the risk, a better stratification of the patients according to the *DDIT4* expression or stratification according other clinic or molecular features could enhance the *DDIT4* value as prognostic biomarker; for example in our work, *DDIT4* overexpression in breast cancer was highly related with the recurrence in the basal subtype (P = 3.8 × 10^−7^) while it had not prognostic value in the HER2-enriched subtype (Fig. 2). In addition, *DDIT4* expression was associated with the molecular risk in acute leukemia in the TCGA cohort (Fig. 1B). Conversely, DDIT4 expression over the median was a strong protective factor in a pool of gastric cancer datasets, although it could not be corroborated with the TCGA data (Fig. 4).

Analysis of protein-protein interaction described that p53 have a key role in the biology of DDIT4 interacting also with several key players in cancer aggressiveness. *DDIT4* gene has a p53 Transcription-Factor Binding Site^15^. A report by Schupp *et al*.^16^, described that p53 is up regulated by *DDIT4* expression under fasting conditions in mice, in addition p53-mediated *DDIT4* expression increase after cisplatin treatment in testicular germ cell tumor-derived human embyronal carcinoma^16, 17^. In the other hand, DIDIT4 exerts feedback control on p53^18^.

The observation that that 17% of breast cancer tumor xenografts develop DDIT4 amplifications in comparison to a low frequency in primary tumors (Figure S1) suggests strongly that DDIT4 activity is important for cancer progression; in the other hand, Bhola *et al*.^19^ described the enrichment of cancer stem cells in TNBC cell lines after treatment with PIK3/mTOR or TORC1/2 inhibitors, it correlates with the worse outcome seen in patients overexpressing DDIT4, mainly in patients with acute myeloid leukemia^19^.

Interestingly, in our analysis with data of 60 cell lines form the Cell Miner Tool Project, mRNA levels of *MTOR* was not associated with drug activity of temosirolimus, everolimus and OS-027, only is associated with rapamicyn activity while *DDIT4* levels were not associated with rapamycin activity (Fig. 7).

Inhibition of *DDIT4* could be a good strategy in cancer treatment. DDIT4^−/−^ cells showed an increased sensitivity to doxorubicin and UV radiation^18^. In a recent work, Potts *et al*.^20^, show that the cyclic depsipeptide didemnin B induce REDD1 loss and mTORC1 activation^20^. In this work a subset of breast, colon, and lung cancer cell lines were selectively sensitive to this drug while ALL cell lines were mostly sensitive. In addition a study in lung cancer cell line (NCI-H460) shows that cucurmin (2 uM) result in down regulation of DDIT4 gene^21^.

In this work we would like to suggest the evaluation of DDIT4 as a prognostic biomarker in malignancies. Evaluation of its involvement in the molecular pathogenesis of acute myeloid leukemias, triple negative breast cancer and other malignancies could identify druggable molecular mechanisms. In contrast to mTOR, DDIT4 levels could be a predictor of response to DDIT4-targeted drugs.

In conclusion, DDIT4 overexpression is related with a worse outcome in several cancer types. Our results are encouraging for the development of DDIT4 inhibitors; it is a rational supported by several *in vitro* studies.

## Methods

### Study characteristics

We evaluated DDIT4 in the outcome of several cancer types in datasets contained in two online platforms: SurvExpress (bioinformatica.mty.itesm.mx/SurvExpress)^22^ and in KM-Plotter (kmplotter.org)^23^. Parameters considered in each online platform were as follows:

SurvExpress

Probes for the gene identifier 54541 (Entrez/GeneID) for DDIT4 with quantile-normalized data were evaluated. The most expressed probe was used in cases of multiple probes for duplicated or alternative probes. Patients in each dataset were divided in two groups according the median of DDIT4 expression. Datasets and endpoints evaluated are described in Table S1.

KM Plotter

The probe 202887_s_at for affymetrix microarray was evaluated. Datasets contained in KM-Plotter and evaluated in this work are listed in Table S1. The entire dataset was split in two groups by the median of DDIT4 expression.

### Survival analysis and Hazard Ratios estimations

In both KM-Plotter and SurvExpress, survival was estimated with the Kaplan-Meier method the Logrank test is used as statistical inference between the two risk groups. The Cox Proportional-Hazards Regression for Survival Data was used to estimate Hazard Ratios. A P < 0.05 was considered statistically significant. There was not adjusting for multiple testing.

### Meta-analysis in SurvExpress datasets

Pooled datasets from SurvExpress were excluded for meta-analysis. Datasets were analyzed individually. Pooled hazard ratios and heterogeneity were analysed using the RevMan program, version 5.3^24^.

### Protein–protein interaction network

The computational tool STRING-9.1 (http://string-db.org) was used to visualize protein-protein interactions between *DDIT4* with relevant gene products in AML and breast cancer, based in data annotated from genomic context, high-throughput experiments, co-expression, and scientific reports. Analysis was done with a high confidence interval (0.7). The green line indicates activation, a red line inhibition, blue line binding, a pink line post translational modification and a yellow line expression.

### Correlation of mTOR drug activities and DDIT4 and MTOR expression

Data transformed to Z-score of mTOR inhibitors and *DDIT4* and *MTOR* mRNA expression was downloaded from the Cell Miner Tool website (http://discover.nci.nih.gov/cellminer/). NSC identifiers were 226080, 606608 and 758664 for rapamycin, 683864 for temsirolimus, 733504 for everolimus and 759660 for OSI-027. Correlation between mRNA expression of 60 cancer cell lines with drug sensitivity in them was done with a regression analysis and correlation coefficient (R-square) was estimated.

### Nanostring analysis and next-generation sequencing

DNA and RNA were extracted from 58 formalin-fixed and paraffin-embedded triple negative residual tumors after neoadjuvante chemotherapy. Gene expression analysis was performed by nanoString and PI3K/mTOR pathway genes were sequenced by next-generation sequencing as previously described^26, 27^. Gene expression values obtained from nanoString were normalized with spike controls, log2 transformed and median centered before the statistical analysis.

### Ethical Considerations

This study involves a reanalysis of gene expression from publicly available datasets.

## Electronic supplementary material

Supplementary infomation
