# Supplementary material for: In silico evaluation of DNA Damage Inducible Transcript 4 gene (DDIT4) as prognostic biomarker in several malignancies
Source: Sci Rep. 2017 May 8;7:1526. doi: 10.1038/s41598-017-01207-3 (PMC5431475; doi:10.1038/s41598-017-01207-3)
Supplement: Supplementary file 1 — Supplementary infomation [file 41598_2017_1207_MOESM1_ESM.pdf]

In silico evaluation of DNA Damage Inducible Transcript 4 gene (*DDIT4*) as prognostic biomarker in several malignancies

Joseph A. Pinto[1], Christian Rolfo[2], Luis E. Raez[3]; Alexandra Prado[1], Jhajaira M. Araujo[1], Leny Bravo[4], Williams Fajardo[4], Zaida D. Morante[7], Alfredo Aguilar[5], Silvia P. Neciosup[1], Luis A. Mas[1], Denisse Bretel[6], Justin M. Balko[8], Henry L. Gomez[5,7]

Affiliations:

1, Unidad de Investigación Básica y Traslacional, Oncosalud-AUNA, Av. Guardia Civil 571, San Borja. Lima 41, Peru.
2, Phase I – Early Clinical trials Unit, Antwerp University Hospital & Center for Oncological Research (CORE), Antwerp, Belgium.
3, Thoracic Oncology Program, Memorial Cancer Institute, Memorial Health Care System, Pembroke Pines, FL, USA.
4, Escuela de Medicina Humana, Universidad Privada San Juan Bautista, Av. José Antonio Lavalle s/n Hacienda Villa, Chorrillos. Lima 09, Peru.
5, Departamento de Medicina Oncológica, Oncosalud-AUNA, Av. Guardia Civil 571, San Borja. Lima 41, Peru.
6, Grupo de Estudios Clínico Oncológicos Peruano, GECOPERU. Lima, Peru.
7, Departamento de Medicina Oncológica, Instituto Peruano de Enfermedades Neoplásicas, Av. Angamos Este 2520, Surquillo. Lima 34-Peru.
8, Vanderbilt Ingram Cancer Center, Vanderbilt University, Nashville, TN 37232-6307.

Corresponding Author:
Henry L Gómez, MD
Departamento de Oncología Médica
Oncosalud-AUNA
Av. Guardia Civil 571, San Borja.
Lima 41, Peru.
Email: hgomezmoreno@gmail.com
Phone: +511 5137900 Ext. 2231.

Table S1.- List of public datasets and endpoints evaluated in the meta-analysis

Cancer type	Platform	Outcome	Dataset	N
**Acute Myeloid Leukemia**	SurvExpress	Overall Survival	Acute Myeloid Leukemia TCGA	168
			GSE12417-GPL96	163
**Breast Cancer**	SurvExpress	Overall Survival	Breast Invasive Carcinoma TCGA	502
			GSE20685	327
			GSE3494-GPL96	502
			GSE11121	200
			GSE1378	60
			GSE3143	158
			GSE19536	110
			GSE25307	577
		Recurrence Free Survival	GSE7390	189
			GSE16391	55
			GSE2034	286
			GSE2990	189
			GSE6532	225
			GSE1456	159
			GSE4922	249
			GSE9195	77
			GSE12093	136
			GSE7378	54
			GSE19615	115
			E-TABM-158	130
			Vant Veer Breast Cancer	78
			van´t Veer - Van De Vijver Nature	295
			GSE45725	340
	KM-Plotter	Overall Survival	E-TABM-365, GSE11121, GSE12093, GSE12276, GSE1456, GSE16391, GSE16446, GSE17705, GSE17907, GSE19615, GSE20194, GSE20271, GSE2034, GSE20685, GSE20711, GSE21653, GSE2603, GSE26971, GSE2990, GSE31448, GSE31519, GSE3494, GSE5327, GSE6532, GSE7390, GSE9195	1117
		Recurrence Free Survival	E-TABM-365, GSE11121, GSE12093, GSE12276, GSE1456, GSE16391, GSE16446, GSE17705, GSE17907, GSE19615, GSE20194, GSE20271, GSE2034, GSE20685, GSE20711, GSE21653, GSE2603, GSE26971, GSE2990, GSE31448, GSE31519, GSE3494, GSE5327, GSE6532, GSE7390, GSE9196	3554
**Glioblastoma**	SurvExpress	Overall Survival	GSE13041 GPL96	218
			GSE13041 GPL570	27
			GSE4412 GPL96	85
			GSE16011	284
			GSE2817	30
			Glioblastoma Multiforme TCGA	538
			Glioblastoma BROAD	50

			GSE7696	84
			GSE42669	58
**Ovarian Cancer**	SurvExpress	Recurrence Free Survival	Ovarian serous cystadenocarcinoma TCGA	578
			GSE9891	285
			GSE32063	40
			GSE32062	255
			GSE30161	58
			GSE17260	110
	KM-Plotter	Overall Survival	GSE14764, GSE15622, GSE18520, GSE19829, GSE23554, GSE26193, GSE26712, GSE27651, GSE30161, GSE3149, GSE51373, GSE9891, TCGA	1582
		Progression Free Survival	GSE14764	1306
**Gastric Cancer**	KM-Plotter	Overall Survival	GSE15459, GSE38749, GSE29272, GSE14210, GSE51105, GSE22377, GSE62254	876
		First Progression	GSE15459, GSE38749, GSE29272, GSE14210, GSE51105, GSE22377, GSE62255	641
	-	Overall Survival	Gastric adenocarcinoma TCGA	208
		Disease Free Survival	Gastric adenocarcinoma TCGA	148
**Melanoma**	SurvExpress	Overall Survival	Skin Cutaneous Melanoma TCGA	16
			GSE22153	57
			GSE19234	44
**Lung Cancer**	SurvExpress	Overall Survival	GSE5123	51
			Lung Adenocarcinoma TCGA	255
			Lung Squamous Cell Carcinoma TCGA	205
			Roepman Lung	148
			GSE3141	109
			GSE37745	196
			GSE42127	133
			GSE4573	130
			GSE19188	64
			GSE17710	56
			GSE31210	226
			GSE30219	264
			Chitale Lung	185
			Bhattacharjee Lung	98
			GSE14814	90
			GSE13213	117
		Recurrence Free Survival	Chitale Lung	185
			GSE17710	56
			GSE37745	196

			GSE8894	138
			Roepman Lung	148
	KM-Plotter	Overall Survival	CAARRAY, GSE14814, GSE19188, GSE29013, GSE30219, GSE31210, GSE3141, GSE31908, GSE37745, GSE43580, GSE4573, GSE50081, GSE8894, TCGA	504
		First Progression	CAARRAY, GSE14814, GSE19188, GSE29013, GSE30219, GSE31210, GSE3141, GSE31908, GSE37745, GSE43580, GSE4573, GSE50081, GSE8894, TCGA	982
**Colon Cancer**	SurvExpress	Overall Survival	Colon Rectal Adenocarcinoma TCGA	151
			GSE28722	125
			GSE24550	77
			GSE17536	177
			GSE12945	62
			GSE30378	83
		Recurrence	GSE14333	290
			GSE28722	125
			GSE24551-GPL11028	160
			GSE24549-GPL11028	83
			GSE17537	55
			GSE31595	37
**Liver**	SurvExpress	Overall Survival	TCGA-Liver-Cancer	422
			Hoshida Golub Liver GSE10143	162
			Liver Hepatocellular Carcinoma TCGA	12
			Hoshida Golub Liver GSE10186	118
		Relapse	TCGA-Liver-Cancer	422
			Tsuchiya Rusyn Liver GSE17856	95
**Kidney**	SurvExpress	Overall Survival	GSE3538	177
			GSE29609	39
			Kidney renal clear cell carcinoma TCGA	468
			GSE33371	23
**Bladder**	SurvExpress	Overall Survival	GSE13507	246
			Bladder Urothelial Carcinoma TCGA	54
			GSE5287	30
			GSE31684	93
**Head and Neck**	SurvExpress	Overall Survival	Head and Neck squamous cell carcinoma TCGA	283
			GSE26549	86
**Prostate**	SurvExpress	Recurrence	GSE40272	98
			Singh Prostate Nature	21
			Taylor MSKCC Prostate	140

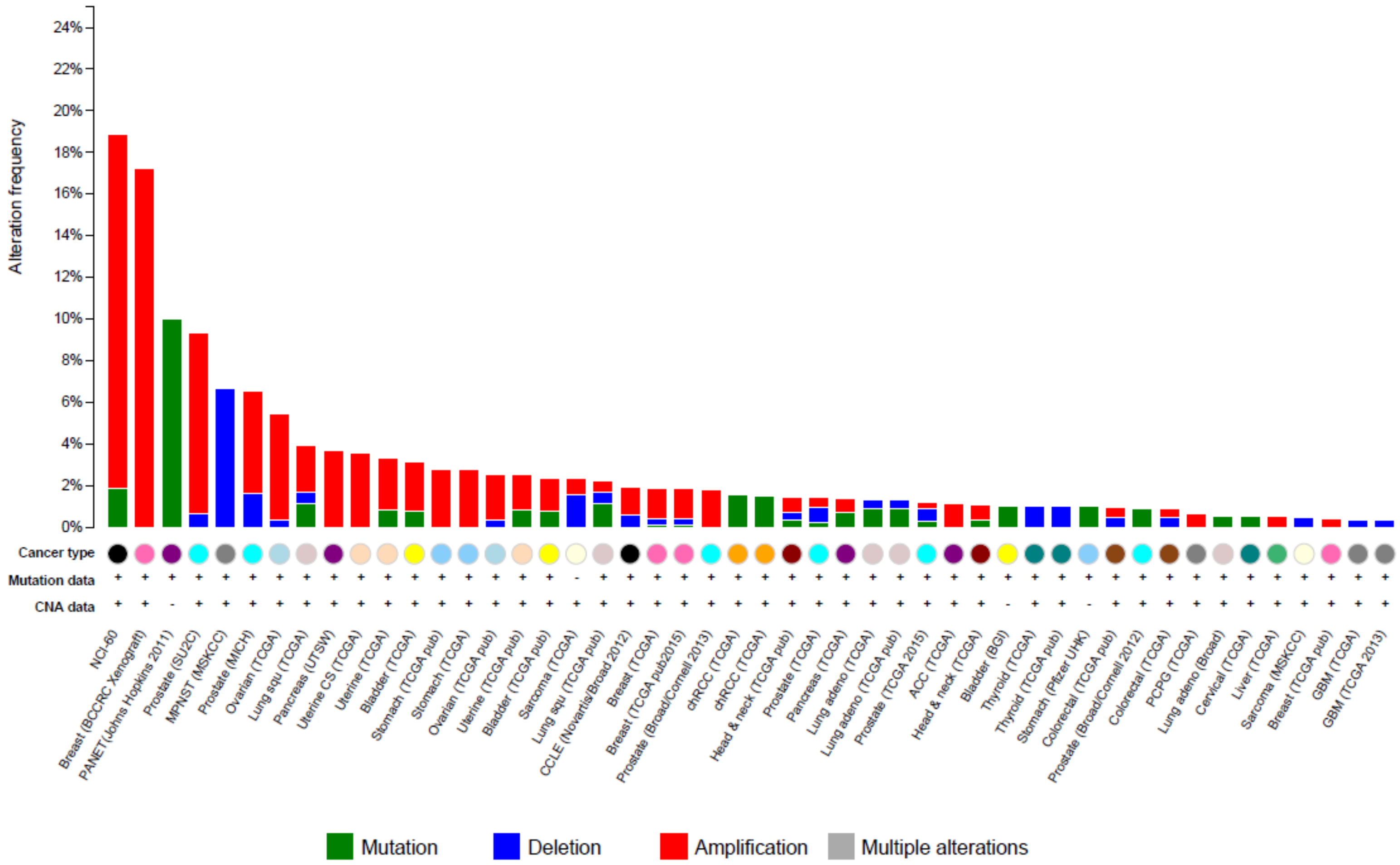

Figure S1.- Frequency of structural DDIT4 alterations in several genomic projects contained in the online plattform cbioportal.org. *DDIT4* amplifications is the most common alteration and were most frequent in cell lines in the NCI-60 project and in breast cancer xenografts

A)

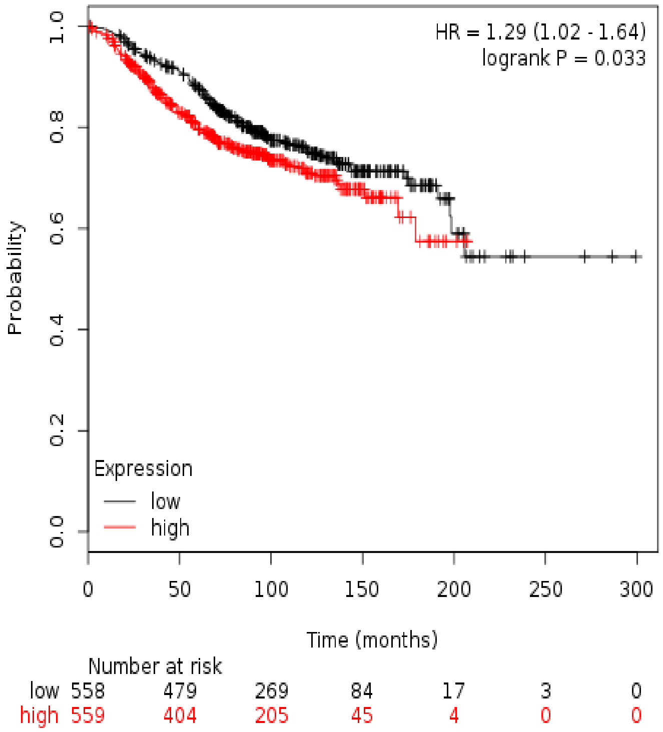

B)

Study or Subgroup	log[Hazard Ratio]	SE	Weight	Hazard Ratio IV, Fixed, 95% CI
GSE1378	-0.16251893	0.37597289	4.9%	0.85 [0.41, 1.78]
GSE20685	-0.02020271	0.21897876	14.4%	0.98 [0.64, 1.51]
GSE3494-GPL96	0.05826891	0.26781177	9.7%	1.06 [0.63, 1.79]
GSE19536	0.19885086	0.3693662	5.1%	1.22 [0.59, 2.52]
GSE3143	0.19885086	0.28626091	8.4%	1.22 [0.70, 2.14]
GSE11121	0.3435897	0.29692389	7.9%	1.41 [0.79, 2.52]
GSE25307	0.42526774	0.13332148	39.0%	1.53 [1.18, 1.99]
TCGA BIC	0.5068176	0.25459377	10.7%	1.66 [1.01, 2.73]
**Total (95% CI)**			**100.0%**	**1.31 [1.11, 1.54]**

Heterogeneity: Chi² = 6.09, df = 7 (P = 0.53); I² = 0%
Test for overall effect: Z = 3.23 (P = 0.001)

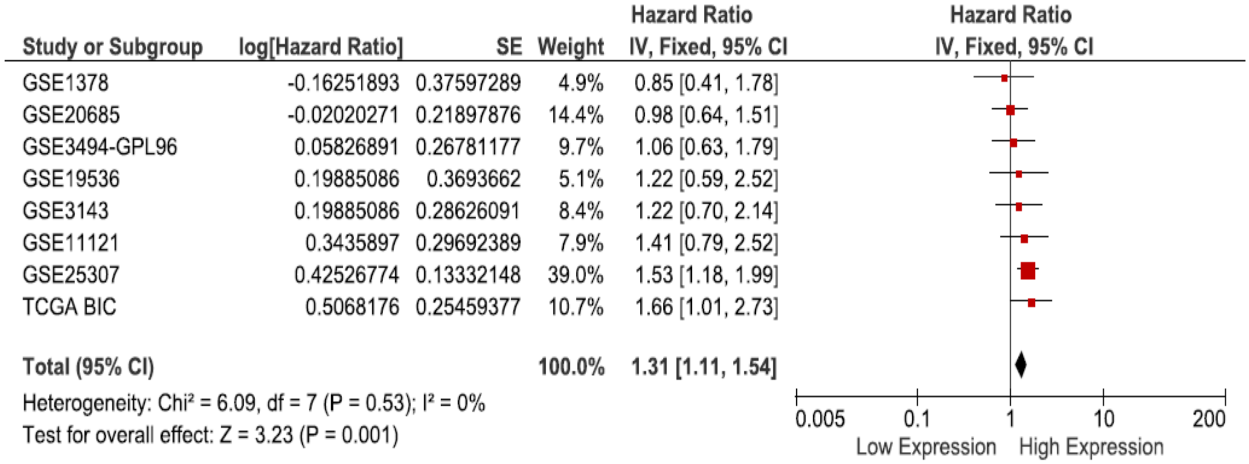

Figure S2.- Influence of DDIT4 expression in overall survival in breast cancer patients. (A) Analysis in KM-Plotter shows that high DDIT4 expression is related with a poor outcome [P=0.033]. (B) A meta-analysis for Overall survival in breast tumors datasets contained in SurvExpress show that DDIT4 expresion over the median increase the recurrence risk in 31%.

A)

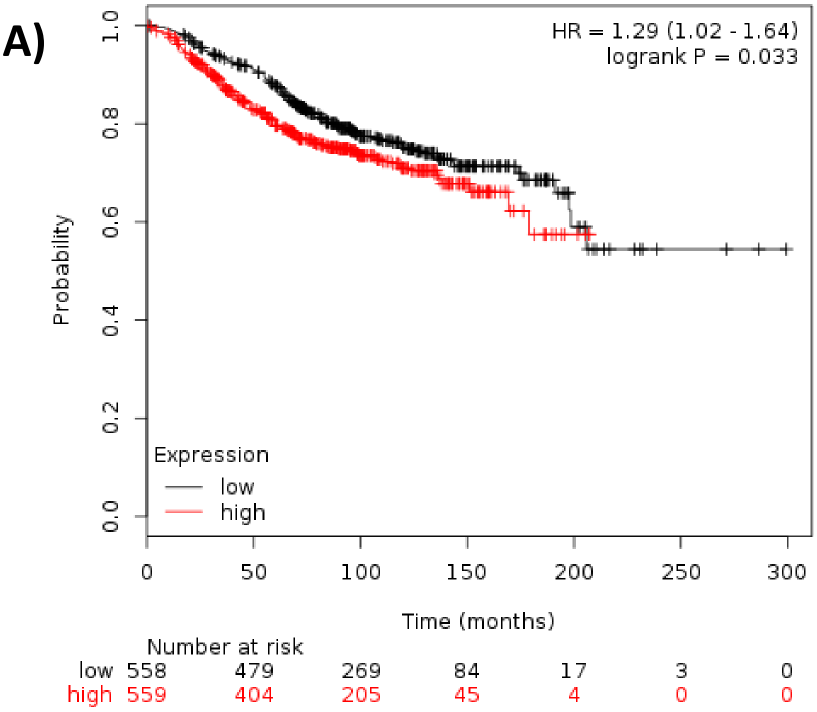

B)

Study or Subgroup	log[Hazard Ratio]	SE	Weight	Hazard Ratio IV, Fixed, 95% CI
GSE1378	-0.16251893	0.37597289	4.9%	0.85 [0.41, 1.78]
GSE20685	-0.02020271	0.21897876	14.4%	0.98 [0.64, 1.51]
GSE3494-GPL96	0.05826891	0.26781177	9.7%	1.06 [0.63, 1.79]
GSE19536	0.19885086	0.3693662	5.1%	1.22 [0.59, 2.52]
GSE3143	0.19885086	0.28626091	8.4%	1.22 [0.70, 2.14]
GSE11121	0.3435897	0.29692389	7.9%	1.41 [0.79, 2.52]
GSE25307	0.42526774	0.13332148	39.0%	1.53 [1.18, 1.99]
TCGA BIC	0.5068176	0.25459377	10.7%	1.66 [1.01, 2.73]
**Total (95% CI)**			**100.0%**	**1.31 [1.11, 1.54]**

Heterogeneity: Chi² = 6.09, df = 7 (P = 0.53); I² = 0%
Test for overall effect: Z = 3.23 (P = 0.001)

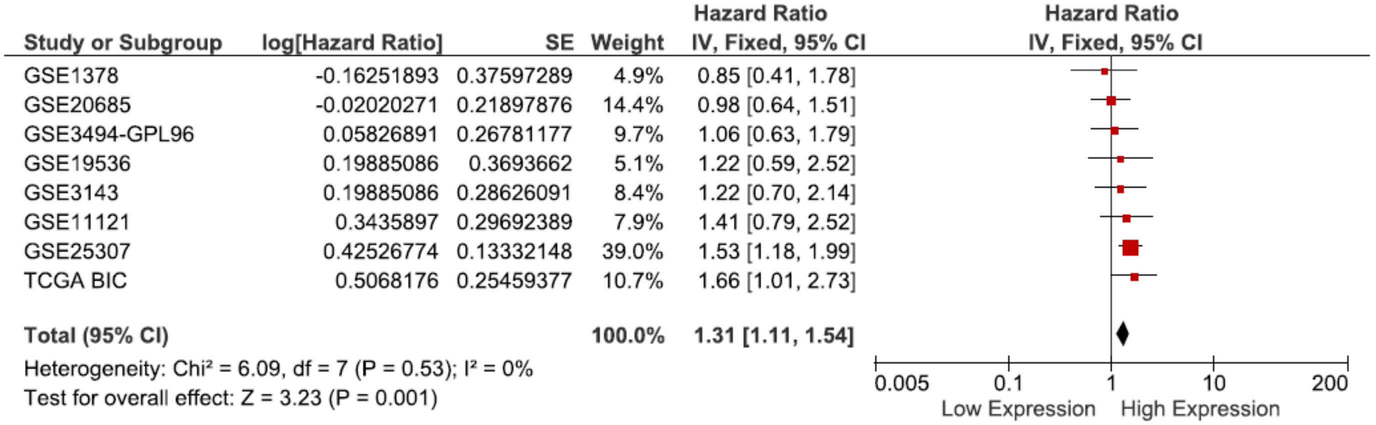

Figure S3.- Influence of DDIT4 expression in overall survival in breast cancer patients. (A) Analysis in KM-Plotter shows that high DDIT4 expression is related with a poor outcome [P=0.033]. (B) A meta-analysis for Overall survival in breast tumors datasets contained in SurvExpress show that DDIT4 expresion over the median increase the recurrence risk in 31%.

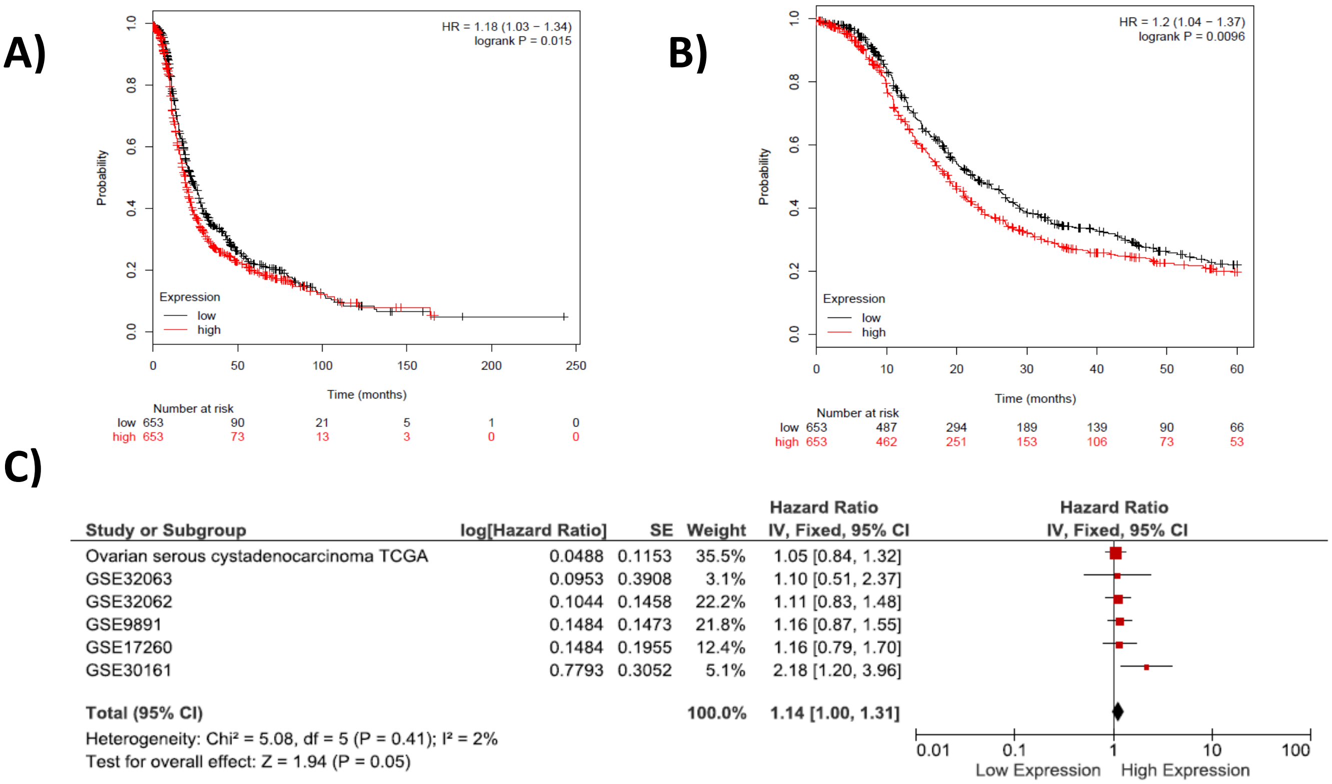

Figure S4.- Influence of DDIT4 expression in progression-free survival in ovarian cancer patients. (A) Analysis in KM-Plotter shows that high DDIT4 expression is related with a poor prognosis [N=1306; P=0.015]. (B) Recurrence-free survival in 5-years censored data. (C) Meta-analysis in datasets of ovarian cancers contained in SurvExpress show statistical trends to a higher risk of relapse in patients with high DDIT4 expression.

Study or Subgroup	log[Hazard Ratio]	SE	Weight	Hazard Ratio IV, Fixed, 95% CI
Skin Cutaneous Melanoma TCGA	0.1398	0.6822	12.3%	1.15 [0.30, 4.38]
GSE19234	0.6931	0.4602	27.0%	2.00 [0.81, 4.93]
GSE22153	0.7561	0.3071	60.7%	2.13 [1.17, 3.89]
**Total (95% CI)**			**100.0%**	**1.94 [1.21, 3.10]**

Heterogeneity: $Chi^2 = 0.68$, $df = 2$ ($P = 0.71$); $I^2 = 0\%$

Test for overall effect: $Z = 2.77$ ($P = 0.006$)

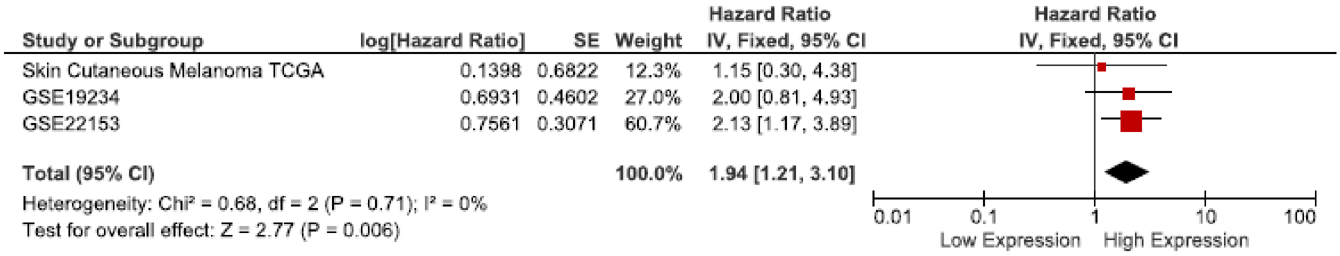

Figure S5.- DDIT4 expression influence the survival in three melanoma datasets from SurvExpress. An expression over the median increases the risk of death in 94% (P=0.006).

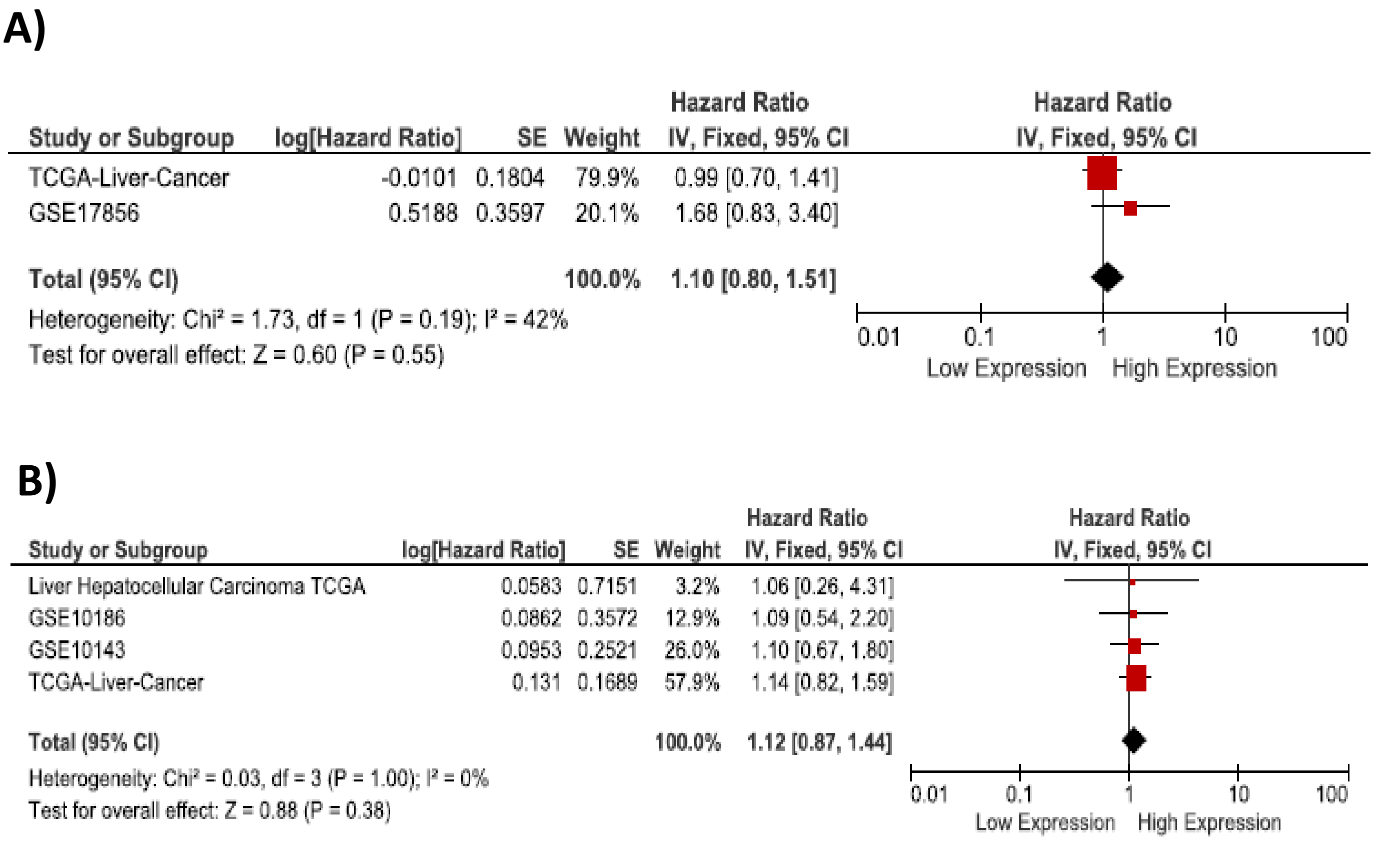

Figure S6.- *DDIT4* was not related with the outcome of liver cancer (evluated in datasets from SurvExpress). In terms of (A) recurrence-free survival or (B) overall survival.

Study or Subgroup	log[Hazard Ratio]	SE	Weight	Hazard Ratio IV, Fixed, 95% CI
GSE3538	0.0677	0.2156	31.4%	1.07 [0.70, 1.63]
GSE29609	0.1222	0.5049	5.7%	1.13 [0.42, 3.04]
Kidney renal clear cell carcinoma TCGA	0.2151	0.1622	55.5%	1.24 [0.90, 1.70]
GSE33371	0.4121	0.4453	7.4%	1.51 [0.63, 3.61]
**Total (95% CI)**			**100.0%**	**1.19 [0.94, 1.51]**

Heterogeneity: $Chi^2 = 0.60$, $df = 3$ ($P = 0.90$); $I^2 = 0\%$
Test for overall effect: $Z = 1.47$ ($P = 0.14$)

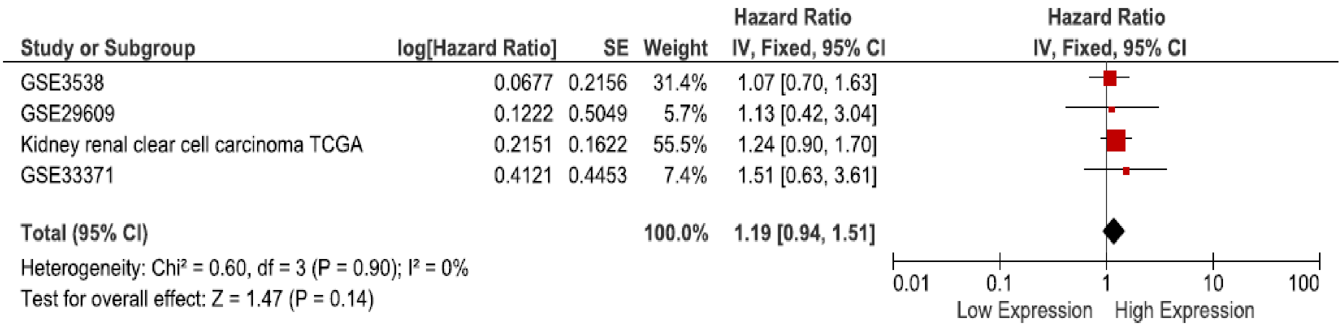

Figure S7.- A expression of *DDIT4* over the median in kidney cancer was not related with the overall survival.

Study or Subgroup	log[Hazard Ratio]	SE	Weight	Hazard Ratio IV, Fixed, 95% CI
GSE13507	0.131	0.2452	45.8%	1.14 [0.70, 1.84]
Bladder Urothelial Carcinoma TCGA	0.157	0.4775	12.1%	1.17 [0.46, 2.98]
GSE5287	0.4447	0.4062	16.7%	1.56 [0.70, 3.46]
GSE31684	0.5822	0.3287	25.5%	1.79 [0.94, 3.41]
**Total (95% CI)**			**100.0%**	**1.35 [0.98, 1.87]**

Heterogeneity: Chi² = 1.43, df = 3 (P = 0.70); I² = 0%

Test for overall effect: Z = 1.82 (P = 0.07)

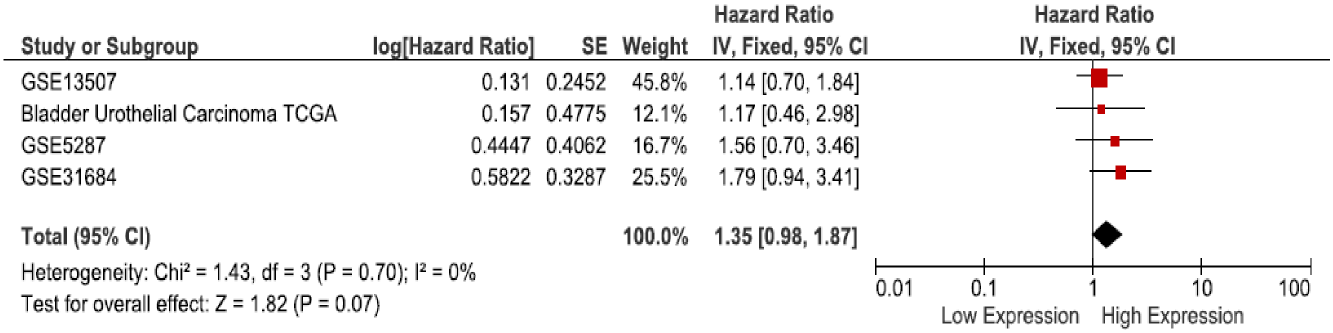

Figure S8.- The meta-analysis of datasets from bladder cancer shown no association with the survival of bladder cancer.

Study or Subgroup	log[Hazard Ratio]	SE	Weight	Hazard Ratio IV, Fixed, 95% CI
GSE10300	0.0583	0.5373	15.9%	1.06 [0.37, 3.04]
GSE26549	0.1222	0.3436	38.8%	1.13 [0.58, 2.22]
E-MTAB-1328	0.4824	0.3175	45.4%	1.62 [0.87, 3.02]
**Total (95% CI)**			**100.0%**	**1.32 [0.87, 2.00]**

Heterogeneity: $Chi^2 = 0.79$, $df = 2$ ($P = 0.67$); $I^2 = 0\%$

Test for overall effect: $Z = 1.29$ ($P = 0.20$)

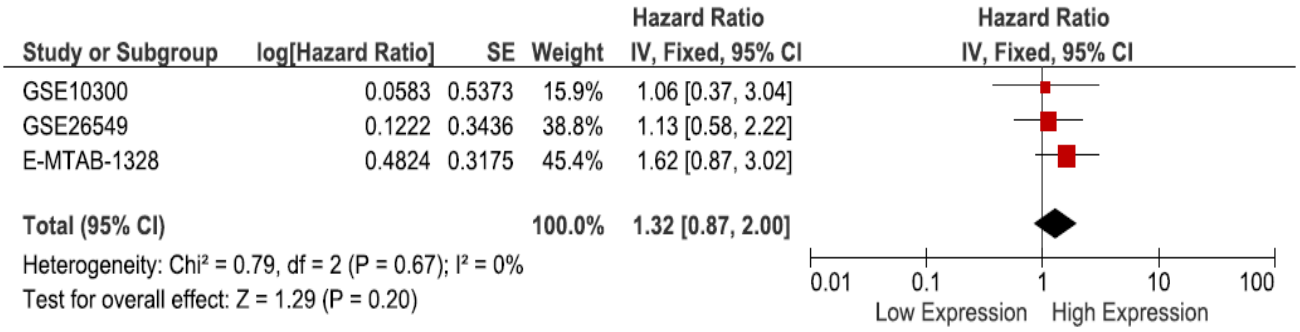

Figure S9.- Meta-analysis of head and neck tumors from Surv express show no relationship with the recurrence-free survival.

Study or Subgroup	log[Hazard Ratio]	SE	Weight	Hazard Ratio IV, Fixed, 95% CI
Singh Prostate Nature	-0.0408	0.7073	12.1%	0.96 [0.24, 3.84]
GSE40272	0.1484	0.4096	36.0%	1.16 [0.52, 2.59]
Taylor MSKCC Prostate	0.4187	0.3411	51.9%	1.52 [0.78, 2.97]
**Total (95% CI)**			**100.0%**	**1.30 [0.81, 2.11]**

Heterogeneity: Chi² = 0.47, df = 2 (P = 0.79); I² = 0%
Test for overall effect: Z = 1.08 (P = 0.28)

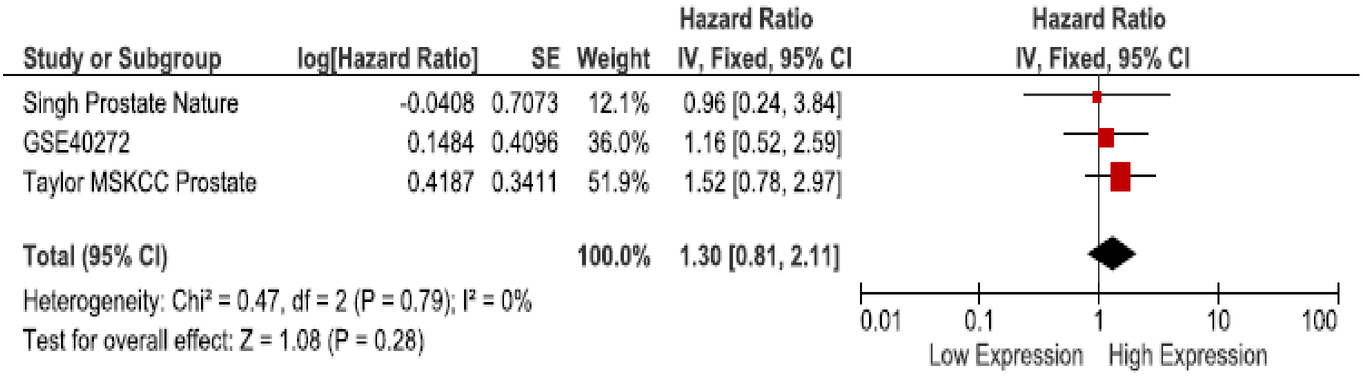

Figure S10.- Meta-analysis of three datasets from SurvExpress shown not association of DDIT4 expression with the recurrence-free survival.
